# A Reduction in the Readily Releasable Vesicle Pool Impairs GABAergic Inhibition in the Hippocampus after Blood–Brain Barrier Dysfunction

**DOI:** 10.3390/ijms25136862

**Published:** 2024-06-22

**Authors:** Kristina Lippmann

**Affiliations:** 1 Carl-Ludwig-Institute for Physiology, Medical Faculty, Leipzig University, D-04103 Leipzig, Germany; kristina.lippmann@medizin.uni-leipzig.de; 2 Grass Laboratory, Marine Biological Laboratory, Woods Hole, MA 02543, USA

**Keywords:** hippocampus, GABAergic inhibition, presynaptic release, short-term plasticity, synaptic vesicle pools, readily releasable pool, blood–brain barrier, stroke, epileptogenesis, gamma oscillations

## Abstract

Major burdens for patients suffering from stroke are cognitive co-morbidities and epileptogenesis. Neural network disinhibition and deficient inhibitive pulses for fast network activities may result from impaired presynaptic release of the inhibitory neurotransmitter GABA. To test this hypothesis, a cortical photothrombotic stroke was induced in *Sprague Dawley* rats, and inhibitory currents were recorded seven days later in the peri-infarct blood–brain barrier disrupted (BBBd) hippocampus via patch-clamp electrophysiology in CA1 pyramidal cells (PC). Miniature inhibitory postsynaptic current (mIPSC) frequency was reduced to about half, and mIPSCs decayed faster in the BBBd hippocampus. Furthermore, the paired-pulse ratio of evoked GABA release was increased at 100 Hz, and train stimulations with 100 Hz revealed that the readily releasable pool (RRP), usually assumed to correspond to the number of tightly docked presynaptic vesicles, is reduced by about half in the BBBd hippocampus. These pathophysiologic changes are likely to contribute significantly to disturbed fast oscillatory activity, like cognition-associated gamma oscillations or sharp wave ripples and epileptogenesis in the BBBd hippocampus.

## 1. Introduction

Blood–brain barrier dysfunction occurs in brain diseases like stroke, brain tumors, traumatic brain injury, or brain inflammation, and often leads to changes in the neural network including overexcitation and disinhibition [1,2,3]. Both may transit into epileptogenesis and deficient cognition-associated network oscillations that constitute a major burden for patients, their relatives, and society [4,5,6,7,8]. To discover therapeutic targets for BBBd-associated dysfunctions and epilepsies, a deeper analysis of pathophysiological changes in neurons is necessary. Previous studies showed that a BBBd after a cortical photothrombosis resulted in epileptogenesis, increased power of theta oscillations and a diminished power in gamma oscillations in the hippocampal area CA1 of rats [9,10]. While it was worked out that more excitatory PCs generate theta resonance behavior and are more excitable [11] contributing towards stronger theta network oscillations, the cellular mechanisms underlying diminished power in gamma oscillations remain largely unknown. Gamma oscillations are essential for information perception, processing, and transfer between brain regions [12,13]. Gamma oscillations are generated by a fast and phasic excitation–inhibition interplay between excitatory PCs and inhibitory fast-spiking parvalbumin-positive interneurons (PV-IN) [12,14,15,16]. The precise timing between PCs and PV-INs determines the oscillatory frequency and thus the correct synchronization for information processing. While PCs predominantly determine the power of theta oscillations due to their intrinsic frequency preference behavior at theta frequency [11,17,18], PV-INs resonate at gamma frequency and are presumably the major clock generator for gamma network oscillations [19,20]. Previous investigations suggested a reduced feedforward and feedback inhibition in CA1 with emphasis on impaired perisomatic inhibition [9]. Perisomatic inhibition onto PCs is majorly provided by PV-INs [21,22]. BBBd following various insults leads to a degradation of the perineural nets of PV-INs [23,24], and may thus impair PV-INs in their proper functions [25,26] up to effects on presynaptic parvalbumin expression [27] and memory [28]. PV-INs are further known to have a high energy expenditure [29,30,31] due to their fast-firing frequencies and fast synaptic transmissions [21]. PV-INs, indeed, contain the largest mitochondria in perisomatic boutons [32] to serve the high energy expenditure for GABA release and vesicular endocytosis [33,34,35]. In post-stroke BBBd, energy levels may be lower, as suggested by spreading depolarizations [9,36,37,38]. PV-INs are also likely to contain ATP-sensitive potassium channels in presynaptic boutons that may open at low ATP levels and diminish neuronal and synaptic activity by an increased potassium efflux [39,40,41,42]. Hence, the aim of this study was to test whether GABAergic release from interneurons, particularly perisomatic interneurons, onto PCs is compromised in the BBBd hippocampus.

## 2. Results

### 2.1. Spontaneous GABAergic Release Is Less Frequent and Decays Faster in CA1 Pyramidal Cells after BBBd

**Figure 1:**
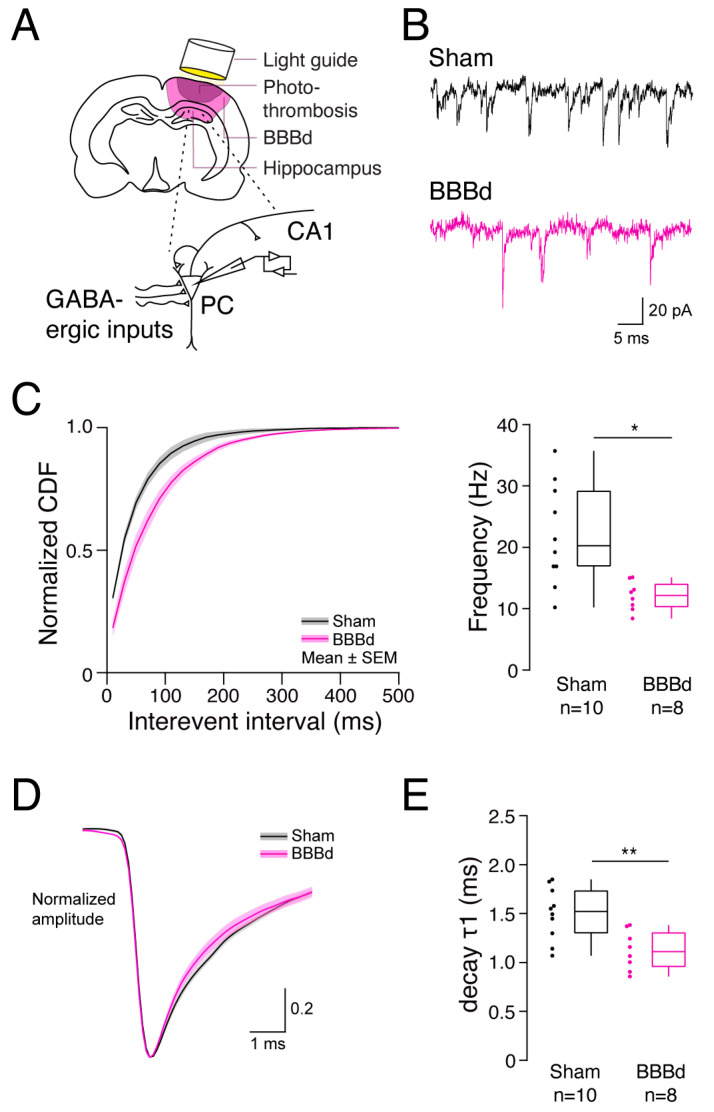
Spontaneous inhibitory GABA release occurs less frequent and decays faster at CA1 pyramidal cells after BBBd. (**A**) Scheme of the experimental conditions. After intravenously injecting Rose bengal (or saline for the sham condition), light was positioned via a light guide behind the Bregma on the right skull of the rat to reliably induce a cortical photothrombotic stroke (dark magenta) and an associated blood–brain barrier disruption (BBBd, light magenta) in the underlying hippocampus. Zoom-in to the hippocampal area CA1 (lower panel). Pyramidal cells (PCs) were whole-cell patch-clamped to record spontaneous miniature inhibitory post-synaptic currents (mIPSCs) while excitatory inputs were blocked. (**B**) Representative PC recordings of a sham (black, upper traces) and a BBBd rat (magenta, lower traces). (**C**) Comparison of the mIPSCs incidence between PCs of sham and BBBd rats, depicted as a normalized cumulative density function (CDF) over the interevent interval (left), and comparing box plots of the mIPSC frequency (right). * denotes a statistical significance of *p* < 0.05. (**D**) Overlay of the mean mIPSCs recorded in PCs of all cells for sham (black) and BBBd (magenta) rats. The mIPSCs were normalized to the trough to enable a comparison of current kinetics. (**E**) The descending part of the mIPSC was fitted with a double exponential fit to extract the normalized amplitudes and time constants (τ) of the mIPSC decay. The boxplot depicts the comparison of the early decay τ1 between sham and BBBd rats. ** depicts a statistical significance of *p* < 0.01.

BBBd was previously shown to lead to hippocampal epileptogenesis due to disinhibition and overexcitation. While the role of spontaneous GABAergic release is not fully understood, it may provide a basal restraint for principal neurons to stabilize networks when no directed activity runs through or is generated at distinct neuronal connections. To dissect which type of inhibition is affected seven days after photothrombosis-induced BBBd (Figure 1A), I conducted whole-cell patch-clamp recordings of PCs in the hippocampal area CA1 and recorded mIPSCs in the presence of tetrodotoxin (Figure 1A,B). The frequency of GABAergic mIPSCs was reduced to 60% in BBBd compared to sham-treated rats (median [interquartile range (IQR)] sham: 20.25 [12.3] Hz vs. BBBd: 12.15 [3.8] Hz, MW, *p* = 0.028, Figure 1C), while the amplitude of mIPSCs remained unchanged (Figure S1A). The rise time of the inhibitory currents (Figure S1B) was not affected by BBBd (Figure S1B,C). However, mIPSCs revealed a faster initial decay time that shortens the inhibitory effect after BBBd (sham: 1.52 [0.44] ms vs. BBBd: 1.11 [0.35] ms, MW, *p* = 0.0062, Figure 1D,E) and may result from an altered expression pattern of GABA_A_R subtypes. The amplitude of the fast component of mIPSC decay was unchanged (Figure S1D), as was the second slower component of mIPSC decay in amplitude and time constant (Figure S1E,F). The major reduction in mIPSC frequency indicates a substantial presynaptic impairment in the spontaneous release of GABAergic vesicles.

### 2.2. Increased Paired-Pulse Ratio in the Fast Gamma Frequency Range of Evoked Presynaptic GABA Release

**Figure 2:**
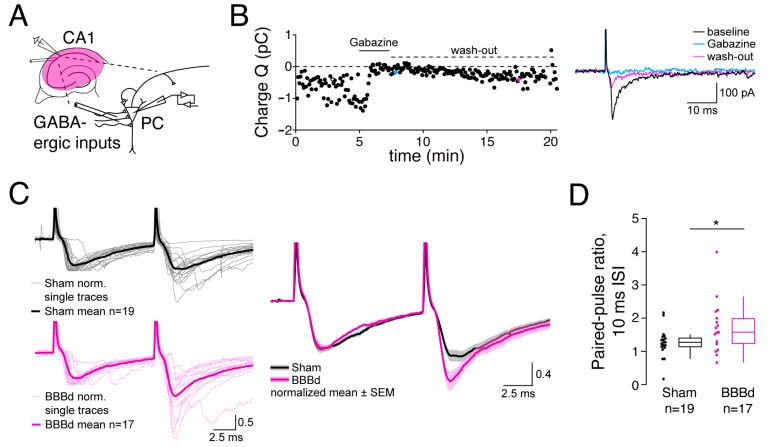
Short-term plasticity of GABAergic inputs is increased after BBBd. (**A**) Schematic drawing of the experimental conditions. Field stimulation-evoked inhibitory GABAergic currents were recorded with whole-cell patch-clamp in PCs in hippocampal area CA1. (**B**) Left: Graph depicts the charge (Q) of evoked inhibitory postsynaptic currents (IPSCs) over time in a patch-clamp recording of a PC. After a stable baseline of 5 min, the GABA_A_ receptor-blocker Gabazine was washed in for 2 min and 20 s, and after the disappearance of the signal, it was washed out again to check for reversibility of the signal. Colored circles indicate example IPSCs shown in traces on the right. Right: Overlay of representative IPSCs during phases of baseline (black), Gabazine application (blue), and wash out (purple). (**C**) Left: Paired-pulse recordings of sham (black, upper traces) and BBBd rats (magenta, lower traces), with an interstimulus interval (ISI) of 10 ms normalized to the trough of the first IPSC. Overlayed thin traces represent the average IPSCs per cell. Thick traces indicate the mean of all evoked IPSCs per group. Right: Comparison of the mean traces per group. (**D**) Comparison of the inhibitory paired-pulse ratios with ISIs of 10 ms in PCs of sham and BBBd rats. Single data points and box plots indicate individual PC recordings. * denotes a statistical significance of *p* < 0.05.

Evoked inhibitory postsynaptic currents (eIPSCs) were then recorded in PCs in the stratum pyramidale to probe whether the evoked GABAergic synaptic transmission is also impaired one week after BBBd (Figure 2A). To first explore whether inhibitory neurons were differentially excitable, the input–output relationships at PCs were quantified, but showed no significant difference between sham and BBBd animals (Figure S2A,B). Subsequent experiments were conducted with two-thirds of the maximum stimulation strength to keep experiments comparable using field stimulation (Figure S2C). For ensuring the GABAergic nature of the recorded inhibitory currents, the GABA_A_-receptor blocker GABAzine (10 µM) was bath-applied, which successfully suppressed the largest portion of the inhibitory input onto PCs. This was partially reversible during wash out (Figure 2B). Single stimuli of similar magnitudes (Figure S2C) revealed the same variability of evoked inhibitory charges, as well as the same failure rate of synaptic transmission after BBBd and in sham-treated rats (Figure S2D,E). In contrast, field stimulation with a paired-pulse paradigm unveiled a stronger paired-pulse ratio in PCs after BBBd (Figure 2C and Figure S2F,G). The paired-pulse ratio was significantly larger, with the shortest inter-stimulus interval (ISI) of 10 ms (sham: 1.27 [0.29] vs. BBBd: 1.58 [0.77], MW, *p* = 0.0486, Figure 2D and Figure S2F,G), i.e., the frequency of fast-gamma oscillations, but not with slower ISIs. This fits with previous observations that showed a reduction in fast-gamma oscillations one-week post-stroke in this model [9]. An increased paired-pulse ratio may indicate a reduced release probability (*p_rel_*) of presynaptic vesicles filled with GABA.

### 2.3. GABAergic Inhibition with a Smaller Readily Releasable Pool after BBBd

**Figure 3:**
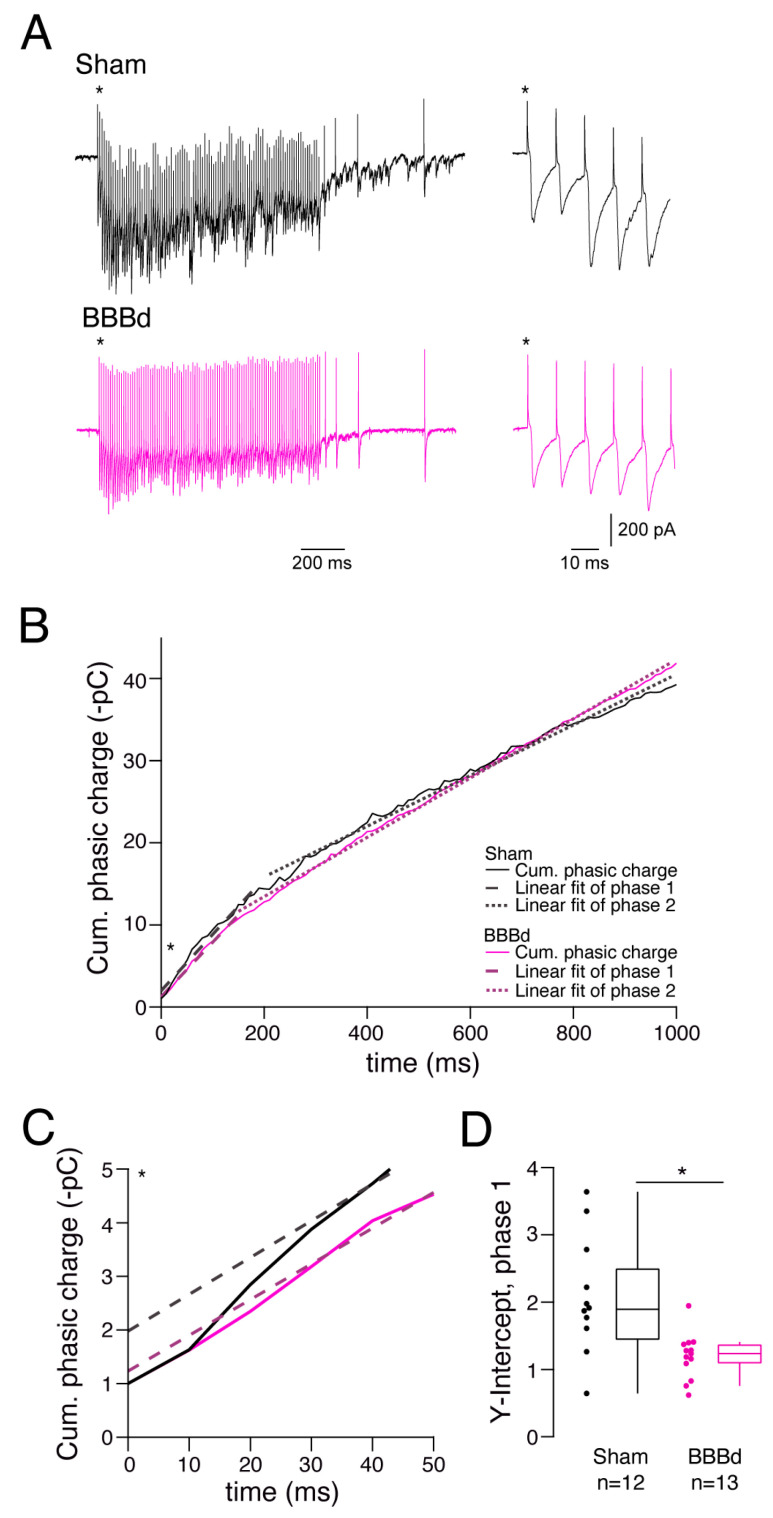
Trains of high-frequency synaptic transmissions reveal reduced readily releasable pools in GABAergic boutons after BBBd. (**A**) Example traces of applied trains to inhibitory synaptic inputs onto PCs. Trains contained 100 pulses with a frequency of 100 Hz, as well as subsequent pulses to determine the recovery after partial vesicle pool depletion during sustained release. (**B**) Exemplary cumulative (cum.) phasic charges plotted over time (solid black lines are sham, and solid magenta lines are BBBd). Dashed lines are linear fits of the first steep phase, and dotted lines are linear fits of the second slower phase of the cumulative charges during the train. Asterisk depicts the zoom-in shown in (**C**). (**C**) Enlarged illustration of the analysis shown in (**B**) that highlights the y-intercept at the beginning of the train. (**D**) Quantification of the y-intercept of the linear fits of the initial train phase, analyzed from sham and BBBd rats. * denotes a statistical significance of *p* < 0.05.

To clarify whether a smaller readily releasable pool or a diminished replenishment rate contribute to the impaired evoked GABA release, high-frequency trains at the compromised frequency of 100 Hz were applied to the inhibitory connections onto PCs (Figure 3A). Train experiments revealed a tonic and phasic component (Figure 3A). Plotting the cumulative charge of the IPSCs, graphs showed a biphasic shape (Figure 3B). To both components, a linear fit was applied that could be extrapolated to the *y*-axis. The slope of the fit provides an estimate of the replenishment rate of the RRP with vesicles from the reserve pool. The y-intercept appraises the size of the number of vesicles in the RRP of GABAergic presynaptic boutons [43,44]. The tonic component revealed no difference for the replenishment rate and RRP size of the typically analyzed second phase of the train. Also, the second phase of the phasic component showed a similar slope and y-intercept (Figure S3B,C), indicating no major change in the replenishment rate and size of the entire readily releasable pool. However, when taking a closer look at the first phase of the train, the linear fits are projected to different y-intercepts (Figure 3C), whereas the slopes remain the same (Figure S3A). The initial y-intercepts may present the number of those vesicles in the readily releasable pool that are tightly docked and thus are ultimately ready for release. After BBBd, the y-intercepts were reduced by 65% from 1.89 [1.06] in sham to 1.23 [0.29] in BBBd rats (MW, *p* = 0.0188, Figure 3D). The recovery rate from the synaptic vesicle pool depletion remained unchanged, indicating no impairment in the refilling of the pools (Figure S3E,F). The stark reduction in ultimately release-ready vesicles may explain the increased paired-pulse ratio, reduced spontaneous release frequency, and a major disinhibition in the hippocampal network.

## 3. Discussion

The present study was designed to probe whether GABAergic release is impaired in the BBBd hippocampus and may thus contribute towards epileptogenesis and dysfunctional gamma oscillations. I found that the spontaneous and evoked presynaptic release of GABA onto PCs is compromised in the BBBd hippocampus, presumably due to a reduction in the number of tightly docked synaptic vesicles in the presynaptic readily releasable pool.

BBBd occurs after diverse insults in human patients that was here exemplarily studied in the model of a cortical photothrombosis, which is reliably inducible and leads to a prominent BBBd in the underlying hippocampus [9,11,23]. The results regarding impaired GABAergic release in this photothrombosis model may be transferable to other insults like traumatic brain injury, brain tumors, or inflammatory brain diseases, and are thus of broader interest [10,23,45,46,47].

Spontaneous GABA release is assumed to provide a steady form of basal inhibition onto PCs to keep the neural network balanced and to serve information processing [48]. It was suggested that both perisomatic cholecystokinin-positive (CCK) and PV-INs share the main load for providing spontaneous release due to no dendritic filtering at the PC soma [48]. Hence, the reduction to about half, as seen in the mIPSC frequency, suggests the existence of a major impairment of spontaneous GABA release in perisomatic interneurons. This may either result from a reduction in the calcium-dependent spontaneous release in CCK-INs, which are microdomain-coupled between calcium channels and sensors, or from the calcium-independent release from nanodomain-coupled PV-INs [48]. Here, fewer tightly docked and ultimately release-ready vesicles could contribute to the diminished mIPSC frequency. The amplitude of the mIPSCs remained unchanged, indicating neither a gross change in the presynaptic vesicle size, nor a stark downregulation or desensitization of GABAergic receptors. However, the faster mIPSC decay provides a shorter inhibitory effect, and may be caused by a slightly different GABA_A_ receptor subtype expression. For example, a somewhat stronger expression of alpha 1 and gamma 1 subunits of GABA_A_ receptors, as previously suggested [9], could fasten the mIPSC decay time and thus shorten the inhibitory GABAergic effect.

To investigate changes in evoked GABA release, inhibitory neurons were activated in stratum pyramidale with a field stimulation to target primarily perisomatic PV-INs. With this approach, it cannot be excluded to also target other inhibitory neuronal subtypes. PV-INs usually show synaptic depression and are phasic synapses [49]. The present paired-pulse ratio in sham rats revealed a slight facilitation, indicative that other interneurons, like CCK-INs, were also activated [50]. In BBBd, the paired-pulse ratio was increased, which suggests that the synaptic depression of PV-INs may be reduced, and the facilitation of other subtypes increased.

Synaptic transmission is thought to involve a two-step priming process of presynaptic vesicles, first into a loosely docked state, and afterwards into a tightly docked state at the presynaptic active zone [51,52]. The functionally observed change in the size of the RRP after BBBd means that fewer vesicles are in the tightly docked state of the RRP. Furthermore, a potential shortage of ATP in perisomatic interneurons due to the photothrombosis-induced BBBd could open ATP-sensitive potassium channels [39,40], and thus hyperpolarize presynaptic boutons due to a constant potassium efflux. Hyperpolarization may decrease spontaneous openings of voltage-gated calcium channels and may thus inhibit the calcium-dependent process of transforming presynaptic vesicles from a loosely docked to a tightly docked and release-ready state. The depressing phasic PV-IN synapse is thought to contain more vesicles in a tightly docked state than a facilitating tonic synapse [51]. Shortly after a stimulus-induced vesicle fusion, a calcium-dependent transient docking of vesicles can occur to refill release sites to serve short-term facilitation [53]. This happens in excitatory synapses between 10–14 ms. PV-INs are optimized for speed and form fast synapses [21]. Hence, the process of transient docking may also be optimized for speed and might occur within 10 ms. The calcium buffer parvalbumin may be reduced in PV-INs due to a degradation in the perineural net [27], as it can occur after BBBd [23]. With its calcium-buffering properties, parvalbumin acts against synaptic facilitation [49,54]. A reduction in parvalbumin might temporarily increase free calcium and thus promote a transient refilling of release sites [53]. A reduction in the RRP of tightly docked vesicles and a reduction in parvalbumin might therefore decrease spontaneous release. This may also apply to the release probability for evoked release and render the phasic synapse into a less phasic/more tonic synapse after BBBd at a fast interstimulus interval of 10 ms (Figure 2C,D), and hence promote disinhibition in the network. To further dissect the interneuronal subtypes and the role of PV-INs, paired recordings of synaptically coupled different interneuronal subtypes and PCs will be necessary in future studies. No changes in the input–output relations onto PCs indicate that the excitability in the axonal compartment before the presynaptic boutons is unlikely to be affected [21], but that indeed the release process of GABAergic vesicles onto PCs is impaired, emphasizing a presynaptic susceptibility. 

The paired-pulse ratio was increased, with an interstimulus interval of 10 ms that reveals an impact onto the generation and modification of fast gamma frequencies at 100 Hz. This fits with previous investigations, where a reduced power in fast- and middle-frequency gamma oscillations were observed in the same BBBd model in in vivo recordings of rats, which also showed epileptic activity [9]. Fast gamma oscillations are thought to contribute to encoding memory [16]. Reduced fast gamma oscillations may thus be one of the key features underlying memory dysfunctions in patients suffering insults associated with BBBd.

Little is known so far about how vesicular pools in interneurons change during physiological activity and which role they play in memory formation. Train stimulation experiments at the fast gamma frequency of 100 Hz revealed a reduction in those vesicles of the readily releasable pool that are presumably tightly docked [51,55] and which are most likely to be released first. A reduction to about half could mean that only half of the neurotransmitter GABA content is released after an incoming action potential in cases under which, in physiological conditions, multiple vesicles would have been released. This may deteriorate strong and fast activity patterns, such as during fast gamma oscillations, synaptic plasticity, or sharp wave ripples.

Experiments in this study were performed on young adult rats, which do not represent the typical age at which insults like strokes or tumors occur. However, patch clamp experiments in the peri-infarct hippocampal slices will become even more difficult in older animals due to increased cell death, membrane instabilities, and enwrapping structures around neurons, preventing access for the patch pipette [56]. The current findings present a snapshot at one-week post-stroke. Future studies will be necessary to further work out whether GABA release recovers in later phases after BBBd, whether it persists, or even whether it aggravates, and therefore deteriorates the processes of epileptogenesis and cognitive dysfunction. Moreover, deeper analyses are necessary with, e.g., super-resolution light and electron microscopy to better understand activity-induced changes in the vesicle dynamics [53], the distances of the vesicles to the active zone membrane [57], and to identify potential target structures for therapeutic approaches to restore inhibition in the BBBd-affected neural network.

## 4. Materials and Methods

### 4.1. Photothrombotic Stroke Induction

For inducing a photothombotic stroke or sham control condition, male adult *Sprague Dawley* rats (8–11 weeks old, purchased from Charles River Laboratories, Wilmington, MA, USA) underwent surgical procedures as described previously [9,10,11,23,37]. In short, ketamine and xylazine (100 and 10 mg/kg body weight (b.w.), respectively) were injected intraperitoneally to deeply anesthetize the rats. For pre-emtive analgesia, animals were treated with metamizole [100 mg/kg b.w. injected subcutaneously (s.c.)] and lidocaine (2% gel on the scalp). Body temperature was controlled via a rectal thermal probe and kept constant at 37 °C via a heating pad underneath the rats. Rats were head-fixed in a stereotactic frame, the skin of the head was scissored, and the muscles were removed from the right scalp. A light guide (∅ = 3.5 mm) was positioned onto the right calvarium (2.75 mm posterior and 2.75 mm lateral from bregma), and either Rose bengal (Sigma-Aldrich, 20 mg/kg b.w., dissolved in saline, animal *n* = 8) or saline (animal *n* = 9, plus 2 naïve rats were added to this cohort) were injected intravenously into the tail vein via a cannula for stroke or sham induction, respectively. The skull was then illuminated for 15 min (150 W halogen lamp, Zeiss KL 1500 LCD, 3E) to activate the photosensitizer Rose bengal or for control conditions. Afterward, the scalp was sutured, and a saline depot was injected s.c. for rehydration (15 mL/kg b.w.). Postoperative analgesia (400 mg metamizole + 4 mL 20% glucose per 100 mL water) was administered for 2 days via drinking water. Animal health was checked daily.

### 4.2. Slice Preparations

Acute brain slices were prepared 6–8 days after surgery. Under deep ketamine/xylazine anesthesia (as described above), rats were transcardially perfused with an ice-cold artificial cerebrospinal solution (aCSF, in mM: 129 NaCl, 21 NaHCO_3_, 1.25 NaH_2_PO_4_, 1.8 MgSO_4_, 1.6 CaCl_2_, 3 KCl, 10 glucose, pH = 7.4, osmolarity 300 ± 5 mOsm, equilibrated with 95% O_2_ and 5% CO_2_) for around 2 min until the blood was fully exchanged with aCSF in the circulation system. After decapitation, brains were rapidly removed and bisected into the two hemispheres. Parasagittal cortico-hippocampal slices (300 µm thick) were obtained from the treated right hemisphere using a vibratome (Leica VT1000S, Leica Biosystems, Wetzlar, Germany). To verify a sufficient photothrombosis through all cortical layers, and thus a BBBd in the underlying hippocampus [9,10,11,23,37]. Post-stroke brain slices were visually checked before recordings on the size of the cortical infarct. Slices were then transferred to a submerged holding chamber with aCSF at 35 °C for 30–40 min and were subsequently kept in aCSF at room temperature until usage.

### 4.3. Electrophysiological Recordings

For whole-cell patch-clamp recordings, slices were transferred to a custom-built fast-perfusion chamber (flow rate: 6–8 mL/min) and held with a platin grid holder onto a SnakeSkin™ Dialysis Tubing (ThermoFisher Scientific, Waltham, MA, USA). This enabled aCSF flow on top of the slice and underneath the membrane to preserve the cell and slice structures as much as possible. For cell visualization, an upright DM6 FS microscope with infrared-differential interference contrast (Leica Microsystems, Wetzlar, Germany) was used. To block ionotropic glutamatergic receptors and isolate GABAergic currents, 4 mM Kynurenic acid (100 mM stock solution solved in 5 mL NaOH and 45 mL double-distilled water) was added to the aCSF during patch-clamp recordings. Whole cell patch-clamp recordings were performed of pyramidal cells (PCs) in the stratum pyramidale of CA1 from the dorsal hippocampus, which was particularly affected by the BBBd [9]. Recordings were conducted at physiological temperature of 35 ± 2 °C under submerged conditions with an intracellular solution containing the following substances in mM: 126 KCl, 4.5 ATP disodium salt, 0.6 GTP sodium salt, 10 HEPES free acid, 1 mM NaCl, 10 EGTA free acid, 4.5 MgCl_2_, and 1.25 CaCl_2_ (310 mOsm, pH of 7.3, no compensation of the liquid junction potential necessary). All substances were either purchased from Sigma-Aldrich (St. Louis, MO, USA) or Tocris Bioscience (Bristol, UK). Patch pipettes were manufactured from borosilicate glass pipettes with a filament (Hilgenberg, Malsfeld, Germany) and pulled with a horizontal puller (Model P-1000, Sutter Instruments, Novato, CA, USA). Voltage clamp recordings were performed with an EPC-10 USB double amplifier and the PatchMaster v2x90.2 software (HEKA Elektronik, Multi Channel Systems MCS GmbH, Reutlingen, Germany). Series resistance was automatically compensated up to 70% when it increased above 10 or 15 MOhm to keep the effective series resistance constant over the recording. To record mIPSCs, 100 nM tetrodotoxin (TTX, Sigma-Aldrich) was bath applied. mIPSCs were recorded for 100 s. To elicit evoked IPSCs in PCs, tract stimulation was performed in the stratum pyramidale with a glass-stimulation electrode and an ISO-STIM 01 DPI stimulator (npi electronic GmbH, Tamm, Germany) triggered by the EPC-10 amplifier. A stimulus-response curve was conducted to receive the maximum inhibitory response and to reduce the evoked IPSC amplitude to ~65% for comparative experiments. To block additional GABAergic currents, 10 µM GABAzine hydrobromide (SR95531 hydrobromide, Cayman Chemicals, Ann Arbor, MI, USA) was bath-applied (Figure 2B). Without GABAzine, about 20–80 repetitions of single stimuli were applied with an interval of 10 s to analyze the charge, coefficient of variation, and failure rate. Paired pulses were induced once per cell for each interstimulus interval (ISIs) of 10,15, 20, 50, and 100 ms every 10 s. Trains of 100 pulses over 100 Hz were applied once per cell. Trains were followed by ‘recovery pulses’ at 25, 50, 100, 300, 1000, and 3000 ms after the end of the train.

### 4.4. Data Analysis

Data analysis was mainly performed with the acquisition and analysis software PatchMaster v2x90.2 and Igor Pro v8.04 (Wavemetrics, Portland, OR, USA), respectively. mIPSCs were analyzed for magnitude and kinetics with NeuroMatic in Igor Pro [58]. Amplitudes, charges, and paired-pulse ratio of the eIPSCs were computed in PatchMaster. The failure rate of eIPSCs was quantified via visual control, whether an identifiable IPSCs could be determined, or no response was visible after a stimulus. Charges were preferred to amplitudes due to the sometimes irregular-shaped IPSCs and better comparability of the effective postsynaptic response. Train analysis was conducted by using a custom-written script in Igor Pro. The tonic and phasic parts of the eIPSCs were extracted. The cumulative charges were computed and linear fits were calculated with the ‘quick fit‘ function in Igor Pro to acquire the slopes and y-intercepts. Data and Igor Pro scripts are available upon request.

### 4.5. Statistical Analysis

Data are presented with single data points and box plots containing the median and the upper (Q3) and lower quartile (Q1). In the text, data are presented with the median and the interquartile range (IQR). Statistical analysis was performed as previously described [9,11]. Data were tested for normality using the Kolmogorov–Smirnov test. Normally distributed data were statistically compared with a Student’s *t*-test (*t*-test). Not normally distributed data were compared with a Mann–Whitney U test (MW). Statistics were performed with the Igor Pro software v8.04. *p*-values below 0.05 were considered to be statistically significant and are denoted with an asterisk.

## Data Availability

The original contributions presented in the study are included in the article/Supplementary Materials, and further inquiries can be directed to the corresponding author.

## Supplementary Materials

The following supporting information can be downloaded at: https://www.mdpi.com/article/10.3390/ijms25136862/s1.
