# Supplementary material for: A Reduction in the Readily Releasable Vesicle Pool Impairs GABAergic Inhibition in the Hippocampus after Blood–Brain Barrier Dysfunction
Source: Int J Mol Sci. 2024 Jun 22;25(13):6862. doi: 10.3390/ijms25136862 (PMC11241665; doi:10.3390/ijms25136862)
Supplement: Supplementary file 1 [file ijms-25-06862-s001.zip › ijms-3062612-supplementary.pdf]

Supplementary Materials

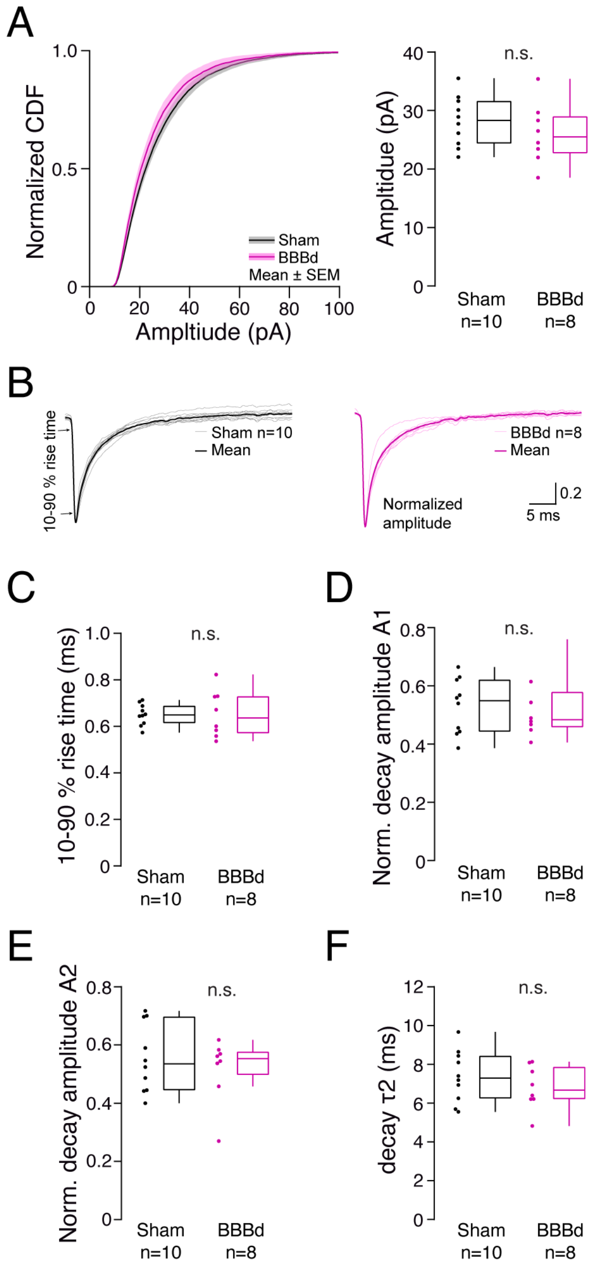

Figure S1 Amplitude, current rise time and slow decay component of mIPSCs in hippocampal PCs are unchanged after BBBd. A Comparison of the amplitude distribution across mIPSCs between sham and BBBd is presented as normalized cumulative density functions (CDF, left graph) as well as median values and boxplots (right graph). **B** Overlay of all recorded and averaged mIPSCs (thin lines) per PCs of sham (gray, left) and BBBd rats (magenta, right). Graphs are normalized to the mIPSC troughs. Thick overlayed traces depict the mean mIPSC of all cells per group. Arrows onto the left graphs indicate the range from which the rise time of the mIPSC was calculated. **C** Comparison of the kinetics of the ascending part of the mIPSCs. The rise time was analyzed between 10 to 90 % of the slope (see B) between PCs of sham and BBBd rats. **D-F** The decaying part of the mIPSCs was fitted with a double exponential fit to extract the normalized amplitudes (A1 and 2) and time constants (τ1 and 2) of the mIPSCs. (D) Quantification of A1, i.e., the amplitude of the fast-decaying component. (E-F) depict the quantification of A2 and τ2, i.e., the slow component of the decaying mIPSCs.

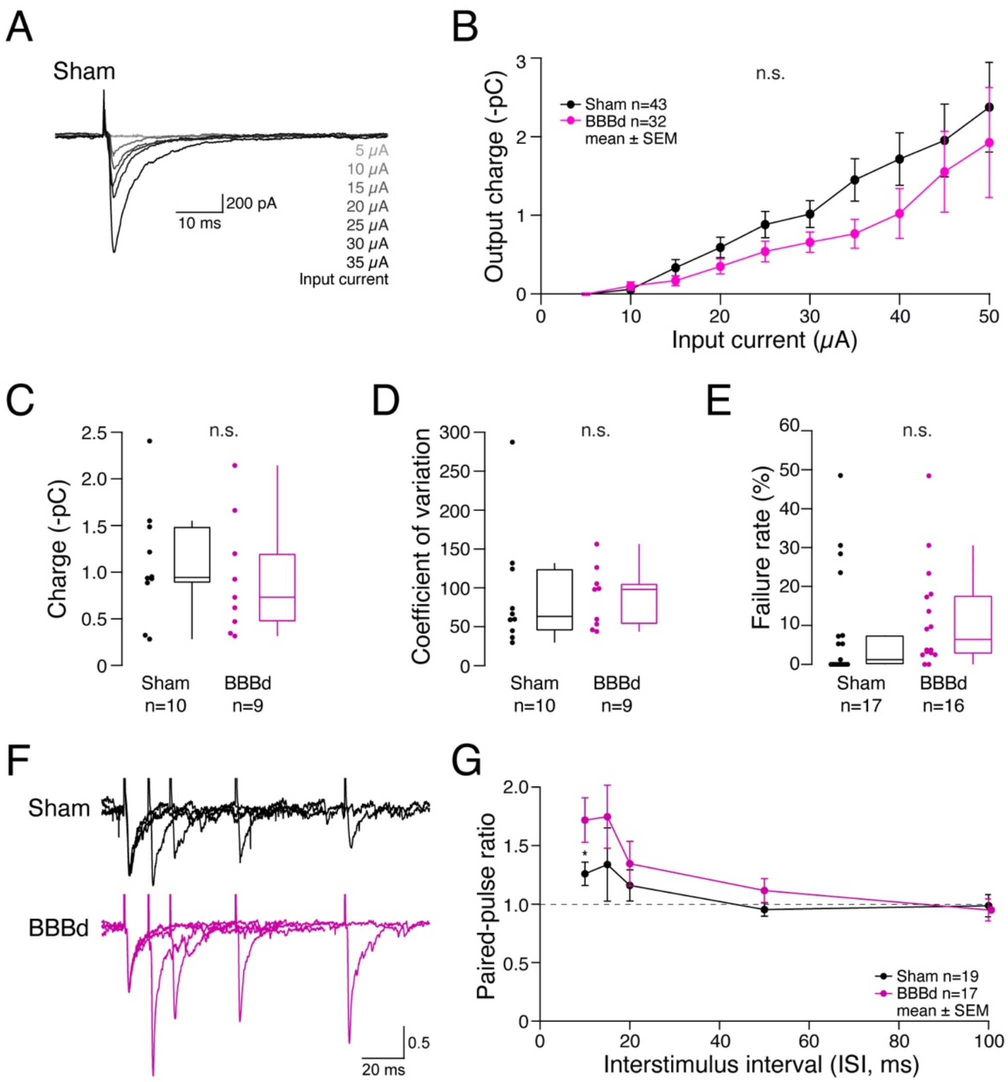

Figure S2. Interneuron excitability, single evoked IPSCs and short-term plasticity with longer ISIs than 10 ms are unchanged after BBBd. A Field stimulation-evoked example IPSC traces of increasing amplitude are shown from a sham rat. Traces are gray scale coded according to the input current strength depicted on the right. **B** Stimulus response curves of sham and BBBd. **C-E** Magnitude of inhibitory postsynaptic charges (IPSQs), coefficient of variation of IPSQs and the rate of synaptic failures are depicted with single data points and boxplots for sham and BBBd rats. **F** Exemplary traces of field-stimulated paired-pulse recordings with ISIs of 10, 20, 50 and 100 ms (sham: black, upper traces; BBBd: magenta, lower traces). The first pulses are overlayed, and the recordings are normalized to the trough of the first IPSC. **G** Quantification of the IPSQ paired-pulse ratios over increasing ISIs (10, 15, 20, 50 and 100 ms) of sham and BBBd. Asterisk indicates a significant difference that is more specifically shown in Fig 2C-D.

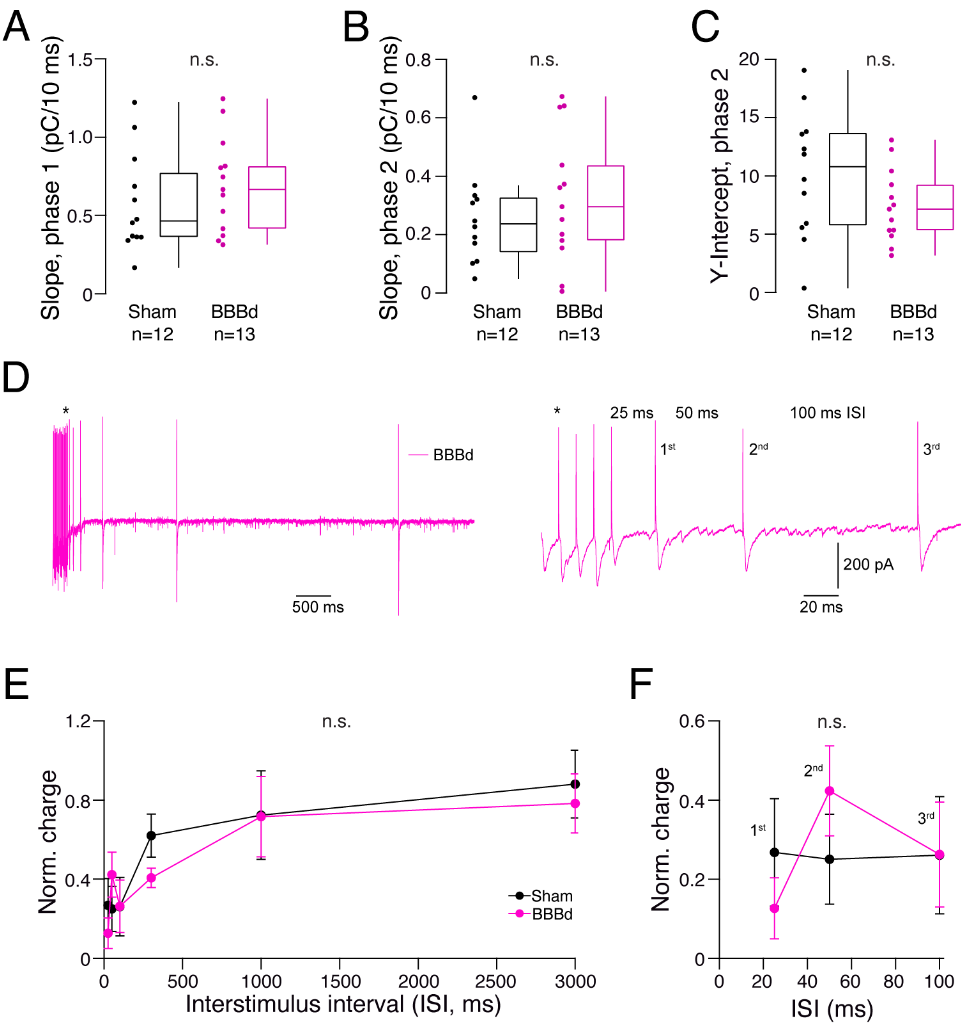

Figure S3. Replenishment of the readily releasable pool and recovery from pool depletion remains intact after BBBd. A-B Comparisons of the slopes of the initial (A) and secondary phase (B) of the cumulative phasic charges from 100 Hz trains that estimate measures of the vesicle pool replenishment. **C** Quantification of the y-intercept of the extrapolated linear fits of the second phase of the cumulative charges from 100 Hz trains, indicative for the number of vesicles in the readily releasable pool. **D** Example trace of the recovery pulses after a 100 Hz train after BBBd. Recovery pulses start with ISIs of 25 ms after the train that were gradually prolonged up to 3000 ms. Only the end of the train is presented but the full range of the recovery pulses (same example as in Fig. 3A). Asterisk depicts the start of the zoom-in shown on the right to visualize the initial three recovery pulses after the train. **E** Quantification of the magnitude of the recovery pulses over time, normalized to the first pulse of the 100 Hz train. **F** Zoom into the comparison of the first three recovery pulses, shown in (E).
